# The delivery challenge of adeno-associated virus vector-based gene therapies for neurological diseases

**DOI:** 10.3389/fnins.2026.1768545

**Published:** 2026-02-26

**Authors:** Alissa Pak, Darcy Wear, Nareh Tahmasian, Jung Yeon Min, Davina Premraj, Rachel Gibbs, Kiah Spencer, Susanna Fang, Thomas Zerbes, Medha Krishnan, Zahra Nasser, Gerold Schmitt-Ulms

**Affiliations:** 1 Department of Laboratory Medicine & Pathobiology, University of Toronto, Toronto, ON, Canada; 2 Tanz Centre for Research in Neurodegenerative Diseases, University of Toronto, Toronto, ON, Canada

**Keywords:** adeno-associated virus (AAV), capsid, gene therapy (GT), neurodegenerative diseases, neurological disease

## Abstract

There is great anticipation that gene therapies can offer solutions to many neurological diseases. Already, much is known about therapeutic targets and how they would need to be manipulated to mitigate disease. For such gene therapies to move to the clinic, potent CNS delivery vehicles are needed. One line of investigation focuses on adeno-associated viruses (AAV) to address this need. In particular, blood–brain barrier (BBB)-penetrant AAV capsids are of interest due to the relative ease of their intravenous administration. This review will introduce this topic and provide an update on recent developments. First, we describe the physical barriers that must be overcome for AAV-delivered gene therapies to reach target cells in the CNS. We then put a spotlight on the natural AAV9 capsid’s inherent propensity to cross the BBB and key lessons learned from its use for delivering a therapeutic payload for the treatment of spinal muscular atrophy. Next, we summarize methods for engineering recombinant AAV (rAAV) capsids with improved brain penetrance, and present *in vitro* paradigms for predicting their capacity to cross the human BBB. We also present strategies for side-stepping the delivery limitations of existing rAAV vectors. Finally, we point toward a few notable clinical studies whose outcomes may advance our understanding of what rAAV-delivered gene therapies can offer to people afflicted with CNS disorders.

## Introduction

The gene therapy field is poised to revolutionize medicine and offer personalized treatment options for a wide range of conditions, including CNS disorders (Ling et al., 2023). AAVs are small, non-pathogenic viruses that belong to the Parvoviridae family (genus Dependoparvovirus) and are widely studied as delivery vehicles for gene therapies. When deployed for this purpose, AAV vectors carry a recombinant DNA payload encoding a therapeutic gene, flanked by inverted terminal repeats (ITRs) essential for packaging and function (Suarez-Amaran et al., 2025). Typically, the payload includes regulatory elements like promoters and polyadenylation signals to control gene expression within the target cell.

Associated with the use of rAAV vector-based gene therapies for neurological diseases are several challenges that must be addressed to ensure successful treatment. These include designing an effective vector payload, avoiding empty capsids or capsids filled with incomplete vector genomes during vector preparation while maintaining high particle yield, choosing an administration regime that limits the host immune response, delivery to the CNS, entering target cells, and ensuring that the gene therapy achieves its therapeutic objective without causing toxicity or unexpected side-effects (Schnödt and Büning, 2017; Higashiyama et al., 2023; Kish et al., 2024).

Interestingly, it is not for a lack of treatment ideas that gene therapies for common CNS disorders are not yet available in the clinic (Li et al., 2023). The etiologies of several of the best-studied dementias are centered on specific proteins—the A*β* peptide and tau protein in Alzheimer’s disease (AD), *α*-synuclein in Parkinson’s disease, TDP-43 in amyotrophic lateral sclerosis and forms of frontotemporal dementias, the prion protein in prion diseases—that can acquire an alternative *β*-sheet-rich folding state as they aggregate and poison cells (Prusiner, 2012). Any method that can reduce the levels of these proteins can be expected to ease disease burden or translate into survival extension.

Often protective variants of the proteins underlying β-sheet-rich depositions in neurodegenerative diseases, or other factors that influence their expression or aggregation, are known. The strongest risk factor for late-onset AD (LOAD) is not Aβ or tau but the apolipoprotein E-ɛ4 variant, which differs from a protective ɛ2 variant in the replacement of two amino acids (Strittmatter et al., 1993). Moreover, the replacement of a single amino acid within the prion protein—evolved in a human tribe afflicted with the disease by spreading it through ritualistic cannibalism—has been credited with the protection against prion diseases (Mead et al., 2009). Consequently, gene therapies may be effective if they instruct brain cells to produce such protective protein variants, either by delivering a payload that codes for them directly or by transducing them with rAAVs coding for a gene editing platform that replaces the respective codons in the endogenous disease genes.

The above are just a few glimpses into a wealth of ideas for how CNS disorders could become treatable with gene therapies. This argument leads to the conclusion that the primary challenge is not the design of effective payloads but the difficulty of delivering them as gene therapies. Moreover, to achieve efficiencies in scale for the population-level treatment of highly prevalent CNS conditions, non-invasive methods of administration are required. The intravenous administration route of rAAV vectors comes with additional adverse effects, such as triggering more robust, undesirable off-target effects, including the transduction of the liver (Brittain et al., 2025), as well as a heightened peripheral immune response (Harkins et al., 2024). The latter confounder is exacerbated by the widespread prevalence of natural AAVs (Earley et al., 2023), which translates into neutralizing antibodies, (NAbs), in addition to challenges posed by the innate immune response (Gross et al., 2022). Pharmacological treatments borrowed from transplant medicine can suppress NAbs and plasmapheresis can remove them (Li et al., 2022). To minimize the innate immune response, avoidance of unmethylated cytosine-phosphate-guanine (CpG) motifs needs to be considered during payload design. This is because Toll-like receptor 9 (TLR9), which acts as an immune sensor of DNA, along with the signaling adaptor MyD88, can induce innate immune responses and CD8 + T-cell responses when exposed to AAV genomes comprising unmethylated CpG motifs. If these motifs cannot be avoided, it may be possible to cloak the AAV genome through the introduction of short DNA sequences that can preempt these unwanted responses by antagonizing TLR9 (Chan et al., 2021). With all these considerations in place, we still need to take into account the payload-encoded transgenes being recognized as foreign, which may force long-term immunosuppressive treatments.

Although the above represent formidable challenges, the increasing availability of clinical data, foremost from the administration of *onasemnogene abeparvovec* (brand name Zolgensma) for the treatment of spinal muscular atrophy, provide hope that these challenges can be overcome (Mendell et al., 2017; Li et al., 2022).

Once in the CNS, rAAV vectors require efficient uptake by target cells. Data from studies with non-human primates have shown that only a subset of brain cells can be targeted upon systemic administration. This shortcoming may represent the single most pressing hurdle to the broad adaptation of rAAV vectors for the treatment of CNS disorders to date. It may be addressed in two ways: by reducing immunological barriers to vector re-administration and/or by improving CNS delivery. This review will focus on the latter of these approaches by briefly introducing barriers to CNS delivery and cellular entry before discussing advances in various domains designed to overcome them, emphasizing reports published in the past 3 years. Since the manuscript is not a Systematic Review, older literature is cited as needed to capture foundational reports of subject matters covered in the review.

## Physical barriers

**Figure 1:**
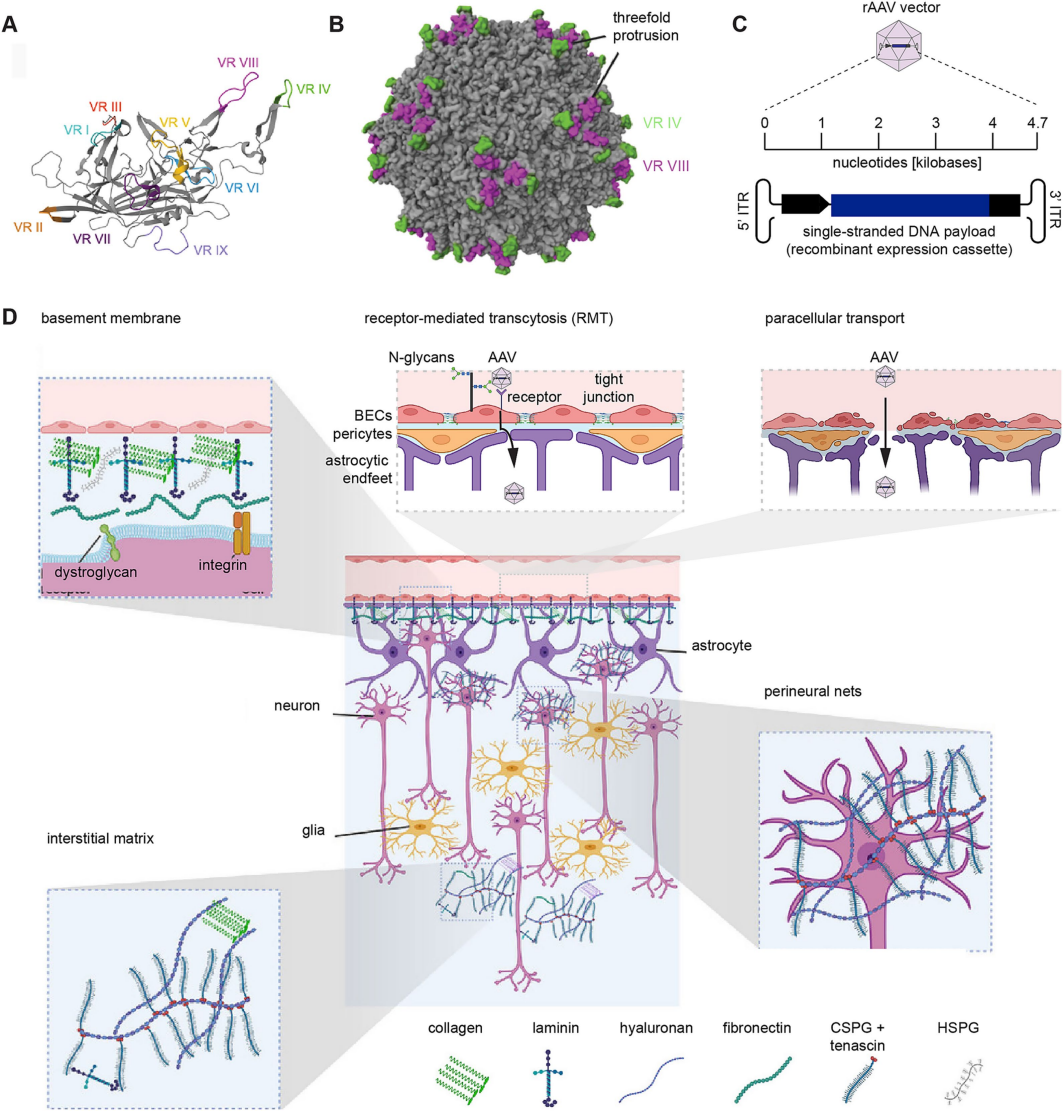
Barriers to the systemic delivery of rAAV vector-based gene therapies for neurological diseases. **(A)** Ribbon diagram of VP1 protein for AAV9, with hypervariable regions VR I-IX labeled. **(B)** 3D structural model of a fully assembled AAV9 capsid (PDB: 3UX1) (DiMattia et al., 2012) created with MolStar (Sehnal et al., 2021), showing the protruding hypervariable domains VR IV (green) and VR VIII (purple). **(C)** Schematic of recombinant AAV payload. **(D)** Physical barrier AAV vectors have to overcome before reaching brain cells. Insets depict the basement membrane, receptor-mediated transcytosis (RMT), paracellular transport, the interstitial matrix, and perineural nets. This panel was created with assistance of BioRender software (https://biorender.com) and modified from an image depicting the extracellular brain matrix (Graber et al., 2024) under the terms of the CC-BY license.

AAV capsids are composed of 60 protomers made of three isoforms of the viral capsid protein (VP1, VP2, and VP3), which assemble into an icosahedral structure. This symmetry is important for maintaining the stability of the virus. Interactions of AAVs with their environment are largely governed by protrusions on the capsid surface. In particular, hypervariable regions (VR) IV and VIII have been shown to mediate docking (Figure 1) (DiMattia et al., 2012). The first hurdle faced by intravenously administered rAAV vectors on their protracted journey to their intended target cells is the BBB, comprised of capillary brain endothelial cells (BECs) that are backed on their basal side by a basal lamina, as well as astroglia and pericytes. The highly restrictive nature of the BBB can largely be attributed to apical tight junctions (TJs) formed by BECs. TJs are composed of occludins (Furuse et al., 1993), claudins (Furuse et al., 1999), and junctional adhesion molecules (JAMs) (Martin-Padura et al., 1998; Jia et al., 2013) and are connected to the actin cytoskeleton through the tight junction protein zonula occludens-1 (ZO-1) (Fanning et al., 1998). TJs serve as selective barriers, allowing the passage of water and small ions while generally restricting the movement of other biomolecules, including toxins and pathogens. Alongside TJs, BECs form transcellular cadherin-based adherens junctions and desmosomes, which are composed of desmoglein and desmocollins (Perl et al., 2024). Combined, these structures hinder the passage of rAAV vectors through the paracellular space.

Consequently, rAAV vectors mainly cross BECs through active transport using receptor-mediated transcytosis (RMT) or caveolae-mediated transcytosis (CMT) (Yang et al., 2025). RMT involves the recognition of viral capsids by specific extracellular transport receptors embedded within the apical plasma membrane facing the lumen. Following recognition, the virus is endocytosed and transported across the BEC, before being released at the abluminal basal plasma membrane. This method of transport has been thoroughly studied with natural AAV vectors, including AAV9, the most brain-penetrant among them (Merkel et al., 2017). Paracellular transport can also occur upon transient or lasting disruptions that impair the structural integrity of the BBB (Adamson et al., 2016), such as osmotic stress following the administration of mannitol (Rapoport, 2000), focused ultrasound (Thévenot et al., 2012), CCL2-induced phosphorylation of *β*-catenin, a building block of adherens junctions (Roberts et al., 2012), or age-related multifactorial weakening of the BBB.

Beyond the BECs, a physical barrier is also posed by the basement membrane (BM). Composed of endothelial and parenchymal layers, synthesized by endothelial cells and glial cells, respectively, the dominant building blocks of this extracellular matrix (ECM) are collagen IV, laminins, nidogens, and perlecan (Xu et al., 2019). While, these components provide structural support for the BBB, they limit the passage of viruses in the systemic circulation to the CNS (Thomsen et al., 2017).

Following its passage through the BBB, a brain-penetrant rAAV vector traverses the extracellular space populated by the interstitial matrix, a loose network ECM largely composed of hyaluronic acid, chondroitin sulfate proteoglycans, tenascin-R, and matrix metalloproteinases. In contrast to many other tissues, the brain’s interstitial matrix lacks fibrillar collagens and fibronectin (Fawcett et al., 2022). As the rAAV vector approaches cells, the interstitial matrix may condense into relatively dense net-like lattices of higher structural organization, termed perineuronal nets (PNNs), that ensheathe cell bodies and proximal dendrites of many neurons (Figure 1) (Fawcett et al., 2022).

After traversing the ECM, rAAVs must then overcome the plasma membrane to enter a target cell. The initial contact with cells may rely on surface glycans (Bell et al., 2011; Shen et al., 2011) and the coreceptor KIAA0319L, also known as the AAV receptor (AAVR), which has broad affinity for a wide range of natural AAV serotypes (Pillay et al., 2016; Summerford et al., 2016). A subset of rAAV vectors also dock to other cell surface proteins, including integrin β1 (ITGB1) (Shen et al., 2015; Havlik et al., 2021).

Once the rAAV vector has engaged in a docking event with a suitable receptor, the rAAV-receptor complex can be internalized by the common clathrin-mediated endocytosis pathway (Bartlett et al., 2000) or a clathrin-independent caveolar/caveosome-like pathway (Bantel-Schaal et al., 2009). Upon internalization, the acidification within entry vesicles contributes to the escape of AAV vectors from them, which may occur within 30 min (Bartlett et al., 2000). Other factors, including GPR108, a member of the G protein-coupled receptor family (Dudek et al., 2020), are required for further processing of the AAV. Mechanisms that govern nuclear uptake, viral uncoating, and expression of the AAV payload are still largely uncharacterized. A subset of AAVs may undergo nuclear uncoating and the subsequent payload expression in as little as 2 h after transduction (Bartlett et al., 2000).

## AAV9 and its use for delivering a gene therapy to SMA patients

To date, much of the attention in the development of gene therapies for CNS disorders has rested on the AAV9 serotype due to its capsid having a relatively high capacity amongst the natural AAVs to cross the human BBB following systemic administration (Merkel et al., 2017). However, the body of CNS work reported with AAV9 and its derivative capsids has revealed considerable spatiotemporal variations in capsid-dependent viral payload expression. For instance, when administered systemically to mice on postnatal day 1, expression of the AAV9-delivered payload could be evidenced in 18% of cortical, 14% of dentate gyrus, and 71% of Purkinje neurons 3 weeks later. These percentages diminish in adult mice (Foust et al., 2009), which mainly exhibit transduction of astrocytes (Gray et al., 2011). Moreover, AAV9 exhibits a high peripheral tropism after systemic delivery and low neuronal transduction in non-human primates (NHPs) (Gray et al., 2011). For instance, in newborn rhesus macaques, AAV9 transduced 0.49% of neurons and 1.86% of astrocytes (Chuapoco M. R. et al., 2023).

Abundant human data have been collected, and valuable lessons have been learned from the use of AAV9 in *onasemnogene abeparvovec* (OA), formerly known as AVXS-101, the first FDA-approved AAV-delivered gene therapy for a CNS disorder. OA represents a self-complementary AAV9 (scAAV9)-mediated gene therapy for spinal muscular atrophy (SMA). SMA is a neuromuscular disorder caused by loss or dysfunction of the survival of motor neuron 1 (*SMN1*) gene, leading to motor neuron degeneration, muscle atrophy, and paralysis. SMA disease severity is modulated by copy numbers of the *SMN2* gene, a paralogous gene that can partially compensate for the loss of *SMN1* function (Lorson et al., 1999).

Early preclinical studies demonstrated that scAAV9 vectors could target motor neurons upon intravenous injection and robustly increase the survival of a mouse model of SMA (Valori et al., 2010), consistent with studies in a cat model of SMA in which motor neuron transduction rates of up to 39% in neonatal cats and 15% in adult cats were observed (Duque et al., 2009). A milestone study reported that postnatal day 1 intravenous injections of scAAV9-hSMN1, a vector that drives the expression of a codon-optimized *SMN1* sequence from a strong phosphoglycerate kinase promoter, increased survival from ~13 days to >340 days, alongside improved weight gain and motor activity readouts (Dominguez et al., 2011). Despite these successes, the path to clinical approval remained fraught with challenges, including a setback of severe toxicity in non-human primates (NHPs) and piglets after a one-time intravenous dose of 2e^14^ viral genome copies per kilogram (Hinderer et al., 2018). The toxicity presented as liver failure in NHPs and ataxia in piglets, which correlated with severe dorsal root ganglia (DRG) sensory neuron lesions. The drivers underlying these inadvertent effects are likely overlapping but non-identical for liver versus DRG. Whereas the liver represents the expected target organ as it evolved to serve as a receptor- and clearance-driven sink (Kaminski et al., 2022), DRG targeting seems to be an exposure-driven neuronal accumulation phenomenon that emerges mainly at high dose. Specific amino acid residues within hypervariable region 1 (VR-I) have repeatedly been shown to contribute to liver targeting, with several single amino acid changes leading to outsized detargeting outcomes (Cabanes-Creus et al., 2021; Xing et al., 2025). Early low-resolution cryo-EM data are consistent with a model whereby the typically buried VR-I domain gets exposed and interacts with AAVR upon acidification in the endo-lysosomal pathway (Meyer et al., 2019). More recently, high-resolution cryo-EM data revealed that the liver tropism can also be modulated by a structural motif within the VR-IV that governs docking to AAVR (Brittain et al., 2025).

In 2019, the FDA approved OA for the treatment of SMA after it was shown that a single intravenous dose of 6.7e13 viral genome copies per kilogram of bodyweight resulted in sustained SMN1 protein expression, and all participants in a Phase I clinical trial (START; NCT02122952) achieved superior motor milestones compared to historical cohorts (Mendell et al., 2017). Long-term follow-up validated that a majority of patients who had received the gene therapy did not require permanent ventilation and retained or gained motor milestones. OA is now approved in more than 50 countries, with over 3,000 patients treated globally, and its indication has recently been expanded by the FDA to older children and young adults albeit with a switch to a single dose intrathecal injection for this older patient cohort (NCT05386680). As expected, the functional benefits are lower when the treatment is administered later, with the highest benefit observed in pre-symptomatic children diagnosed through newborn screening (Chand et al., 2021; Servais et al., 2024). Although these are remarkable accomplishments that were achieved well ahead of their time, safety concerns remain, including adverse events (AEs) in almost half of the patients, which can be serious in approximately 6% of patients (Chand et al., 2021; Servais et al., 2024). To improve safety, the objective to maximize on-target efficacy must be balanced against the adverse costs of off-target effects, including hepatotoxicity (Chand et al., 2021). Data from SMN1-overexpressing mice document significant protein aggregation, resulting in the secondary sequestration of small nuclear ribonucleoproteins, leading to abnormal splicing and alterations to the transcriptome (Van Alstyne et al., 2021). Consequently, it would be desirable to keep the SMN1 expression at physiological levels. One way to achieve this is to swap the artificial promoter used in OA with the natural promoter derived from the *SMN1* gene (Xie et al., 2024). Moreover, it is apparent that there is broad heterogeneity in the treatment benefit (Reilly et al., 2022). Finally, the long-term challenges faced by children who received OA remain uncertain. This uncertainty has many causes, including limited data for projecting the degree to which the efficacy may fade over time as the *SMN1* gene may get epigenetically silenced or diluted in dividing cells.

To address some of these challenges, it would be preferable to work with AAVs whose propensity to enrich in the liver or dorsal root ganglia is low and whose propensity to transduce the CNS is high. It also would be desirable to have access to a repertoire of AAVs that can transduce specific cell types in the CNS. The conceptually obvious but technically challenging answer to attaining these goals is to engineer rAAV variants that overcome these limitations.

## Methods to improve brain penetrance of natural AAVs

**Figure 2:**
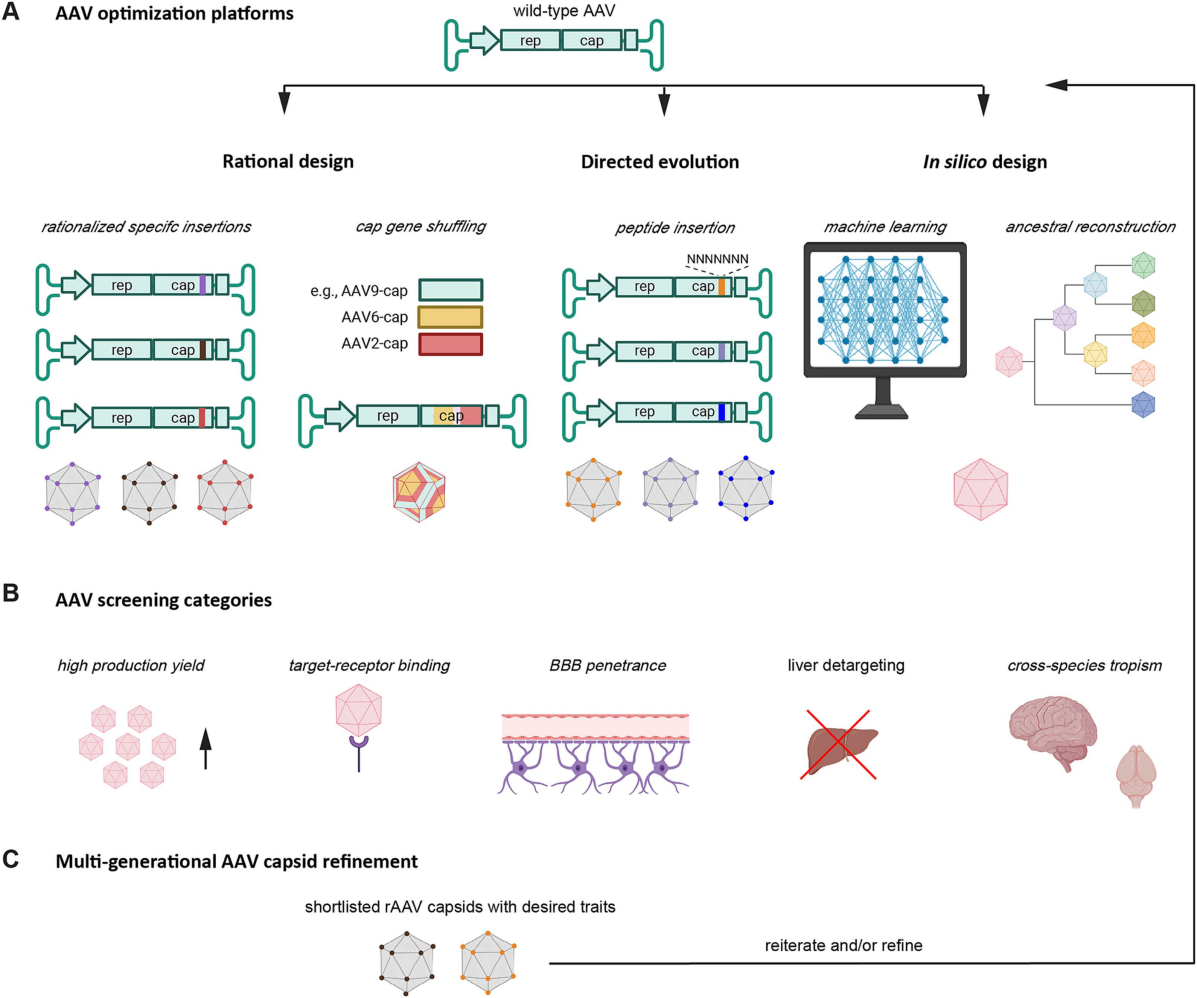
Methods for improving and refining rAAVs. Illustration depicting the main rAAV capsid engineering strategies. **(A)** First-generation rAAV capsids are typically derived from natural AAV capsids through methods that can broadly be categorized as rational design, directed evolution, and *in silico* design approaches. Rational design may include the hypothesis-driven insertion of specific receptor recognition sequences or the shuffling of pre-existing *cap* gene sequences. Directed evolution may take the insertion of a library of short sequences into specific hypervariable regions. *In silico* design may rely on computational power through machine learning or ancestral reconstruction. **(B)** A critical component in the optimization pipeline is the selection of rAAV capsids with desired traits. The screening for capsid tropism can follow the rAAV design process (rational design) or be integral to the selection process (directed evolution). **(C)** The optimization of several traits may require iterative rAAV capsid refinement. Increasingly, the trend is to capture several desired traits concomitantly. This shift saves time and is better equipped to deal with a reality whereby desired properties of rAAV capsids do not manifest in a strictly orthogonal manner. Portions of this image were created with assistance of BioRender software (https://biorender.com).

There are three dominant categories that current methods for optimizing rAAV capsids can be grouped into: rational design, directed evolution, and *in silico* design (Figure 2). This section will only briefly showcase how these approaches are implemented. The reader is pointed toward recent reviews for a more in-depth discussion of this topic (Becker et al., 2022; Ingusci et al., 2024; Xu et al., 2024).

The rational design approach commonly relies on targeted modifications to a rAAV capsid through site-directed mutagenesis, for instance, the swapping of a surface-exposed tyrosine or threonine with less polar phenylalanine or valine residues, respectively (Markusic et al., 2010; Gilkes et al., 2021), or the systematic replacement of one or a more amino acids (Li et al., 2012). Despite the low-throughput nature of this strategy, impressive advancements have been achieved with it. For instance, a comparison of AAV9 variants observed in NHPs drew attention to rare variants coding for Y527 and S533 (VP1 numbering) which map to three-fold protrusions known to engage in interactions with cellular receptors. This finding led to the AAV9. HR capsid, one of the first liver-detargeted AAV variants (Wang et al., 2018). Rational design can also involve swapping domains that confer known interactions (Albright et al., 2018).

Some studies use pre-selected libraries of insertion sequences. For instance, AAV9P1, which displays improved tropism for astrocytes, was generated through the screening of a fixed library of peptide insertions (Kunze et al., 2018). A peptide insertion at AA585-586 (VR-VIII) that included the integrin binding sequence RGD, followed by a leucine residue, enhanced transduction of astrocytes. AAV9P1 was subsequently shown to require the avβ8 integrin receptor for transduction. These studies also showed that the RGD motif is stronger than the NGR integrin binding motif present in AAV9 for promoting astrocyte transduction (Kunze et al., 2018). In addition to peptide insertions and modifications, rational design can also involve chemical modifications of amino acids on the surface of AAV capsids. The incorporation of an unnatural amino acid carrying an azido group can be achieved by first inserting a UAG stop codon into the capsid coding sequence, then co-expressing a prokaryotic tRNA and the corresponding tRNA synthase during rAAV assembly to force the mammalian translation machinery to recognize the stop codon as a regular codon and incorporate the azido-lysine moiety. When followed with chemo-selective conjugation of moieties to the azido group, this system can retarget a capsid to a different cell surface receptor (Kelemen et al., 2018). For instance, conjugation of folic acid directed capsids to the folic acid receptor, which is highly expressed in certain cancer cells (Puzzo et al., 2023). However, this type of bioconjugation is limited by the size of the moieties that can be added and the complexity and heterogeneity of bio-conjugated rAAV capsids.

Increasingly, computer-generated docking algorithms are being used to assist in rational design. In one approach, design choices are informed by modeling the binding of ligands to their respective receptors (Abramson et al., 2024). For instance, structural predictions assisted by modeling with AlphaFold2 were deployed to direct a rAAV capsid to human integrin αVβ6 (Vu Hong et al., 2024).

Directed evolution is an engineering method in which a diverse library of AAV capsid variants is generated, followed by a selection process that isolates and identifies capsids with desired properties. Advantages of direct evolution methods are that they require little understanding of the structure–function relationship of the capsid, facilitating the discovery of variants with novel properties. Recent efforts to improve directed evolution have focused on optimizing screening platforms. A well-known platform makes use of Cre recombination-based AAV targeted evolution (CREATE) (Deverman et al., 2016). In this implementation, once the AAV capsid library has been generated, it is administered to animals expressing the Cre recombinase in target cells to identify cell type-specific capsids (Chan et al., 2017). Tropism Redirection of AAV by Cell-type-specific Expression of RNA (TRACER) requires payload expression for selection. By also incorporating cell-type-specific promoters, TRACER can identify AAV variants that express in a cell type of interest (Nonnenmacher et al., 2021). Another screening platform, termed directed evolution of AAV capsids leveraging *in vivo* expression of transgene RNA (DELIVER), is compatible with extremely diverse capsid libraries and shares with TRACER the need for payload expression (Tabebordbar et al., 2021).

Candidate rAAV capsids identified by directed evolution undergo target deconvolution to identify their mechanisms of transduction using methods such as cell microarray technology (Shay et al., 2024). In this approach, which operates like an inverse transfection, DNA encoding human cell surface receptors are spotted onto an array, with reporter cells cultured on top. The screen captures the binding of a rAAV capsids, equipped with a detection label, on cells that express a specific receptor (Shay et al., 2024). A screen may also reveal other rAAV interactors, as was the case for interleukin-3 interacting with AAV9, a finding that may suggest a mechanism by which intravascular AAV9 modulates immune responses (Shay et al., 2024).

More recent additions to the toolbox of capsid optimization strategies are *in silico* design approaches, which rely on computation to overcome the limits of conventional rational design approaches. This includes methods that build on ancestral reconstruction, which uses phylogenetic analyses of natural AAVs to reconstruct evolutionary lineages of capsid sequences with improved tropism. This method has led to the identification of several capsid serotypes currently undergoing clinical testing. For example, the reconstructed ancestral AAV Anc80L65 was shown to have increased CNS tropism in a comparison with AAV9 (Hudry et al., 2018). In addition, machine learning (ML) is increasingly complementing rAAV capsid engineering efforts. ML models can be trained on experimental data to design diverse rAAV capsid variants with desired properties. For instance, deep learning has been applied to design thousands of rAAV capsid variants that remain viable for viral packaging (Ogden et al., 2019; Bryant et al., 2021). By harnessing the power of ML, multiple desirable properties can be optimized simultaneously. A recently developed ML approach, Fit4Function, showcased the systematic engineering of multi-trait rAAV capsids. So far, this approach has been used to generate candidates with high production yields and liver-specific tropism (Eid et al., 2024).

Finally, the optimization of rAAV vectors can draw on hybrid approaches. For example, a rAAV identified by directed evolution can undergo rational engineering or shuffling of capsid coding sequences with the aim to confer the best properties in more than one assessment category (Albright et al., 2018). In a hybrid approach that combined elements of rational design (the targeting of a specific receptor) with a randomization strategy, a four-step strategy was employed for *de novo* capsid generation. First, a library of AAVs comprising randomized 7-mer peptides at a hypervariable region was generated. Subsequently, capsid variants that bound target receptors immobilized on magnetic beads were enriched *in vitro*. Next-generation amplicon sequencing of the respective capsid payloads identified the specific 7-mer peptides that conferred binding. Finally, promising AAV candidates were subjected to secondary *in vivo* and *in vitro* validation. In a first proof-of-concept study of this approach, AAVs were generated that interact with lymphocyte antigen 6A (LY6A) or 6C1 (LY6C1) proteins present on mouse BECs to improve CNS penetrance in mice (Huang et al., 2023). In a more recent study, the same team targeted the well-characterized human transferrin receptor (TFR1), which is expressed on human BECs (Qin et al., 2023). The resulting rAAV capsid, known as B-hTFR1, was subsequently tested in knock-in mice expressing the human extracellular domain of TFR1 and had 54 times higher transduction levels compared to AAV9. Importantly, B-hTFR1 targets the receptor’s apical domain and does not compete with the highly abundant human transferrin. The successful generation of B-hTFR1 highlights the potential of rational design to develop more efficient rAAV capsids for human therapy.

## rAAVs of interest for CNS applications

A productive line of investigation designed to improve the brain tropism of AAV9 for adult mice was based on the CREATE directed evolution method. These efforts led initially to AAV-PHP.B (Deverman et al., 2016) and subsequently to AAV-PHP.S for the transduction of the peripheral nervous system (PNS) and AAV-PHP.eB for CNS transduction, respectively (Chan et al., 2017). AAV-PHP.eB was shown to transduce 69% of cortical and 55% of striatal neurons. A secondary screen built on AAV-PHP.eB led to AAV.CAP-B10 and AAV.CAP-B22 capsids. These capsids were also selected based on their lower propensity to target the liver and improved brain tropism in marmosets (Goertsen et al., 2022). Remarkably, improvements in the CNS transduction of marmosets was subsequently shown to translate poorly to infant rhesus macaques (Chuapoco et al., 2022). To address this shortcoming, the same team followed up with a screen using a multi-species selection strategy, initially screening in common marmosets, then testing shortlisted rAAV variants in two Old World primate species, including the rhesus macaque (*Macaca mulatta*). This effort resulted in a capsid with good CNS tropism in macaques, fittingly termed AAV.CAP-Mac (Chuapoco M. R. et al., 2023). A detailed follow-up investigation of the CNS tropism of AAV.CAP-Mac in several Old World monkey species validated the enhanced specificity of the capsid for neurons over astrocytes (Chuapoco M. R. et al., 2023) while highlighting species- and age-related complexities.

Strain-specific differences in transduction efficiency are a well-known challenge in AAV research. For example, potent brain tropism was observed upon intravenous delivery of AAV-PHP.B or AAV-PHP.eB in C57BL/6J but not in BALB/cJ mice (Hordeaux et al., 2018). Genomic studies across mouse strains identified Ly6a as the capsid-interacting protein expressed on the surface of BECs, with genetic knockout of *Ly6a* in C57BL/6J BECs reducing AAV-PHP.eB binding affinity by 50%. In addition, a frequent *Ly6a* single-nucleotide polymorphism (SNP) was observed to manifest in high expression of this protein in C57BL/6J strains and low expression in BALB/cJ mice (Arboleda-Velasquez et al., 2019; Hordeaux et al., 2019). Notably, no Ly6A homolog exists in primates (Loughner et al., 2016), providing a straightforward explanation for why PHP.B and several of its derivatives exhibit poor CNS penetrance in NHPs.

The performance of a select few capsids that exhibit exceptional transduction of mice but not NHPs has prompted targeted screens for rAAV capsids directly in NHPs. One rAAV capsid with high BBB penetrance that emerged from a TRACER directed evolution screen in NHPs is 9P801 (Voyager Therapeutics) (Nonnenmacher et al., 2022). Intravenous administration to adult Cynomolgus macaques revealed that 9P801 has a low tendency to target the liver and DRG, paired with a high brain cell tropism that is predominantly targeting neurons. To our knowledge, no receptor candidate has been reported for this capsid, thereby making it difficult to predict its efficacy and safety profile in humans.

One way to circumvent safety concerns associated with the orphan receptor status of capsids is to specifically screen for rAAV capsids that bind to human orthologs of known receptors. For instance, mouse-potent capsid 9P31, also developed by Voyager Therapeutics, has a 400-fold greater capacity to transduce the brain than AAV9 (Nonnenmacher et al., 2021) and docks to carbonic anhydrase IV (CA-IV) (Shay et al., 2023; Zhang et al., 2024; Lin et al., 2025). Although this protein is present in rodents and humans, species differences in the primary sequence of CA-IV meant that 9P31 only interacts well with the rodent protein, limiting the use of this capsid to pre-clinical studies. To address this limitation, another team reported recently on the *in vivo* selection of a capsid library in mice engineered to express human CA-IV. To limit the search space, the capsid library had been *in vitro* pre-selected for binding to human CA-IV. Interestingly, top performing capsids selected from the *in vitro* screen performed poorly when administered to CA-IV humanized mice. Nonetheless, the shortlisted capsid that emerged from this search, termed AAV-hCA4-IV77, was validated to targets human CA-IV and achieved a 100-fold higher CNS transduction than AAV9 (Lin et al., 2025). Results from this study suggest that weaker receptor-binding may be important for efficient receptor-mediated transcytosis. Importantly, these performance discrepancies highlight the importance of increasing translatability through the use of “humanized” models.

Even when a rAAV vector binds to a receptor whose sequence is highly conserved across species, transduction rates for brain cells can still differ greatly in cross-species comparisons (Table 1). An example represents the capsid VCAP-102, which was derived from AAV9 through a TRACER screen (Moyer, 2024). When administered systemically, VCAP-102 transduced approximately fivefold more neurons in adult NHPs (20–30%) than adult mice (5.5%) and nearly tenfold more astrocytes (60–77%) in African green monkeys than in adult mice (8%) (Moyer et al., 2025).

**Table 1:** rAAV capsids of interest for human CNS applications sorted by publication date.

Capsid	Month/Year	Parent capsid	VP1 modification	Primary CNS tropism	Receptor	% of brain cells transduced in mice	% of brain cells transduced in NHPs	Human paradigms	Ref
AAV9	N/A	N/A	N/A	Neurons and astrocytes	AAVR, galactose	14–71% of cortical neurons and Purkinje cells, 60% of lumbar spinal cord neurons in neonatal mice, >64% of astrocytes in lumbar spinal cord of adult mice	0.49% of neurons and 1.86% of astrocytes in young green monkeys.	Clinically approved for the treatment of SMA	Foust et al. (2009) and Mendell et al. (2017)
AAV-BR1	06/2016	AAV2	N587Q substitution, GNRGTEWDA insertion at AA588	BECs	**	Cortical, hippocampal, and cerebellar BECs	**	**	Körbelin et al. (2016), Kawabata et al. (2023), and Rasmussen et al. (2025)
Olig001	09/2016	AAVs 1, 2, 6, 8, 9	Capsid shuffling	Oligodendrocytes, few astrocytes and neurons	**	>70% oligotropism except for cerebellum	** 90–94% specificity to oligodendrocytes in the striatum and corpus collosum in adult rhesus macaques	** rAAV-Olig001-ASPA is currently in Canavan disease Phase 1/2 trial	Powell et al. (2016) and Francis et al. (2021)
AAV9P1	02/2017	AAV9	RGDLGLS insertion at AA585-586	Astrocytes	ITGAV: ITGB8, AAVR, galactose	>90% cortical astrocytes	**	*78.87% of cultured astrocytes	Kunze et al. (2018) and Bauer et al. (2022)
AAV-MG1.1 and 1.2	08/2022 06/2025	AAV9	LMTPPKTTS or TEPPKTTS insertion at AA588-589	Excitatory neurons	**	Initially reported to target >80% of microglia. Now shown to target >95% of hippocampal excitatory neurons	**	**	Lin et al. (2022) and Cao et al. (2025)
AAV-BI30	04/2022	AAV9	NNSTRGG insertion at AA588-589	BECs	**	84% of BECs	**	72–96 fold increase in hBECs *in vitro*.	Krolak et al. (2022)
9P801	10/2022	AAV9	**	Neurons	**	**	>90% of neurons and >40% of brain cells in permissive regions, >20% of brain cells in non-permissive regions in adult cynomolgus macaque	**	Nonnenmacher et al. (2022)
AAV-BR1N	06/2023	AAV2	GNRGTEWDA insertion at AA588	Neurons	**	Cortical, hippocampal, and cerebellar neurons	**	**	Kawabata et al. (2023)
AAV. CAP-B10	01/2022	AAV-PHP.eB	DGAATKN substitution of AA452-460	Neurons	**	* ~ 37% of neurons.	4-fold increase in neuronal transduction compared to AAV9 in marmosets but not macaques	**	Goertsen et al. (2022) and Stanton et al. (2023)
AAV. CAP-Mac	01/2023	AAV9	LNTTKPI insertion at AA588-589	Neurons and some astrocytes	LRP6, AAVR	**	47–60% of neurons and rarely in astrocytes in infant rhesus macaques 33–51% of neurons, 3–21% of astrocytes in adult green monkeys	45-fold increase in iPSC neurons relative to AAV9	Chuapoco M. et al. (2023) and Shay et al. (2024)
PAL2	01/2023	AAV9	EVGPTQGTVR insertion at AA588-589, substitution at 587–588	Neurons and astrocytes	**	**	80% of motor cortex neurons, 50% of striatal neurons, 40% of thalamic neurons, and 55% of astrocytes in adult cynomolgus macaques	**	Stanton et al. (2023)
AAV-X1.1	05/2023	AAV9	GNNTRSV insertion at AA588-589, substitution at AA452–458	BECs and neurons	LRP6, AAVR	~82–85% of GLUT1 + cells with 90–92% specificity	~45 fold increase in neuronal transduction with 98% specificity in neonatal rhesus macaques.	22–44% of BECs *in vitro*	Chen X. et al. (2023) and Shay et al. (2024)
VCAP-102	05/2023	AAV9	** HDSPHK insertion at AA453-460	Neurons and astrocytes	ALPL, AAVR, galactose	~13% of neurons, ~14% of astrocytes, 38% of brain cells in motor cortex	20–30% neurons and 60–77% astrocytes in adult African green monkeys	**	Hoffman et al. (2023) and Moyer et al. (2025)
BI—hTFR1	12/2023	AAV9	7-mer insertion at AA588-589	Neurons and astrocytes	hTRF1	54% cortical, 32% striatal, 68% thalamic neurons, 80% astrocytes in cortex, striatum and thalamus.	**	** Transduction of hBECs *in vitro*	Qin et al. (2023)
VCAP-Gen2	05/2024	VCAP-102	** Mutagenesis of exposed surface loop	Neurons and astrocytes	ALPL, AAVR, galactose	** ~ 60% of brain cells in adult mouse cortex	25–66% of neurons 87–97% of astrocytes in cynomolgus macaque	**	Moyer (2024) and Moyer et al. (2025)
STAC-BBB	05/2024	**	**	Neurons	**	34–66% of brain cells	Neuronal transduction ranging from ~26–65% in adult cynomolgus macaque	>90% in iPSC neurons	Tiffany et al. (2024), Chou et al. (2025a), and Chou et al. (2025b)
AAV-DB-3	05/2025	AAV1	7-mer insertion at AAV590	Neurons	**	Neurons in deep brain structures following direct intracerebral injection	45% of medium spiny neurons in striatum of adult rhesus macaque	Only intraventricular data reported	Leib et al. (2025)

Transduction efficiencies refer to IV administration unless otherwise specified. N/A = not applicable, AA = amino acid. BECs = brain endothelial cells. ICV = intracerebral ventricular. IV = intravenous. ** = information not available. * = information estimated from graphs.

Several factors may contribute to such species-specific differences in tropism, including trivial reasons such as brain sizes and differences in virus dosages administered. More complex causes may include differences in neutralizing antibodies, the expression levels and binding affinities of ortholog primary receptors (Shay et al., 2013)—alkaline phosphatase (ALPL) in this case (Moyer et al., 2025)—as well as species divergence in co-receptors, and downstream processing pathways that govern the expression of the rAAV-delivered payload. Even small changes can have profound effects on the tropism of engineered AAVs. For instance, the AAV-BR1 capsid which had been shown to exhibit preferential tropism for BECs (Körbelin et al., 2016) was turned into a preferentially neuron-targeting AAV-BR1N capsid by exchanging a single amino acid (from a glutamine to asparagine at position 587) within its surface VR-VIII region (Grimm and Nonnenmacher, 2023; Kawabata et al., 2023).

Similar considerations apply to age-dependent changes in rAAV tropisms. It is well established that the expression of proteins, including possible receptors, changes with age within and across species. Additionally, age-related morphological changes to the brain can markedly impact the distribution and clearance of AAV vectors. In neonates, a larger ventricular-to-parenchyma ratio allows broader vector distribution following CSF delivery, while there is increased retention of vectors in the parenchyma and reduced spread in aged mice, a phenomenon likely exacerbated by deregulation of the waste clearance (glymphatic) system in aged brains (Choudhury et al., 2016). Additionally, aged brains exhibit heightened seroprevalence of neutralizing antibodies against AAVs, with the lowest prevalence in humans aged 3–17 for all serotypes (Perocheau et al., 2019). Moreover, humans and mice exhibit enhanced BBB permeability with age. For instance, the frequency and lengths of tight junction breaks are increased in the cortex and cerebellum of mice (Goodall et al., 2018). With regard to receptor docking, 20-months-old rodents have been documented to display loss of striatal *N*-glycans and *N*-acetylated heparan sulfate disaccharides, which are critical for AAV9 binding (Raghunathan et al., 2018).

Recently, a VCAP-Gen2 capsid was derived from VCAP-102 in a secondary mutagenesis screen which monitored brain transduction and liver detargeting, leading to several-fold improvements in both selection categories. Specifically, in cynomolgus macaques, VCAP-Gen2 was reported to transduce 12 and 43% of neurons and 51 and 60% of astrocytes in the cortex and thalamus, respectively (Moyer, 2024).

Similar data in NHPs are not yet reported for the aforementioned BI-hTFR1 capsid. However, in knock-in mice, which were made to express the human transferrin receptor, this capsid was reported to transduce 54 and 71% of neurons and 86 and 92% of astrocytes in the cortex and thalamus, respectively (Qin et al., 2023).

STAC-BBB, developed by Sangamo Therapeutics, is another promising capsid that exhibits excellent cross-species brain-wide tropism. This capsid preferentially targets neurons and exhibits 100-fold improved de-targeting of peripheral tissues relative to AAV9 (Chou et al., 2025a; Chou et al., 2025b). Screened from a library of over 100 million capsids, STAC-BBB was reported to achieve 644-fold enrichment in the macaque brain compared to AAV9.

For the treatment of some CNS conditions, it is desirable to target specific CNS cell types as opposed to broadly transducing all brain cells. For instance, specifically targeting microglia could pave the way for gene therapies that take advantage of their unique roles as resident immune cells of the brain (Rosario et al., 2016). In 2022, it was reported that AAV-MG1.1 and MG1.2 capsids may be useful in this regard, as they seemed to target >80% of the microglia surrounding the site of intracerebral injection (Lin et al., 2022). A recent study cast doubt on this conclusion (Cao et al., 2025). Specifically, the authors proposed that the microglia-directed tropism may have been an artifact of a screen that relied on microglia-based Cre expression. Consistent with this interpretation, when this limitation was experimentally lifted, the cell-type specificity of the capsids shifted from microglia to excitatory neurons.

In addition, AAV-X1.1 is a promising rAAV that primarily targets BECs, with potential applications for the treatment of stroke, as well as vascular derivatives of Alzheimer’s disease and related dementias (Bryant et al., 2023). In adult mice, AAV-X1.1 transduced 90–92% of endothelial cells across the brain (Chen X. et al., 2023).

Finally, Olig001, designed by shuffling sequence elements of several natural AAV capsids (AAVs 1, 2, 6, 8, 9) and directed evolution in rats, has >95% tropism for striatal oligodendrocytes (Powell et al., 2016). The high level of oligotropism exceeding >70% (except for the cerebellum) makes this capsid a candidate for delivering gene therapies for demyelinating diseases such as Canavan Disease, a fatal CNS condition in children caused by loss-of-function mutations within the human oligodendroglial aspartoacylase (*ASPA*) gene (Francis et al., 2021).

If no capsids with a desired cell type-specific tropism are available, cell type specificity of AAV-delivered gene therapies can still be achieved by limiting payload expression. The classical solution to this challenge is to drive the expression of payload-encoded transgenes from cell-type-specific promoters. Efforts to limit the size of these promoters to accommodate other payload elements are ongoing and have led to minimal promoters, including promoters that can drive preferential expression in astrocytes (gfaABC(1)D, 681 bp), oligodendrocytes / Schwann cells (MAG, 1451 bp), or neurons (p546, 546 bp) (Chornyy et al., 2025). A flurry of recent reports showed that this goal can also be attained by embedding cell type-specific short enhancer elements within the payload. Specifically, by diving deep into single-nuclei transcriptomic and epigenomic data, with or without the help of machine learning, several teams reported specific enhancer elements which restricted expression to cell types that are central to neurodegenerative disease etiologies, including cortical or striatal interneurons, cortical neurons, pyramidal neurons, or spinal motor neurons (Furlanis et al., 2025; He et al., 2025; Kussick et al., 2025). In light of the many biological intricacies that govern capsid tropism, it seems unlikely that capsids with desired properties can be designed purely based on deep understanding of the pertinent biology. Rather, advances in this area need to be paired with investments in the development of *in vitro* paradigms that closely mimic the human brain.

## Predicting human BBB penetrance

**Figure 3:**
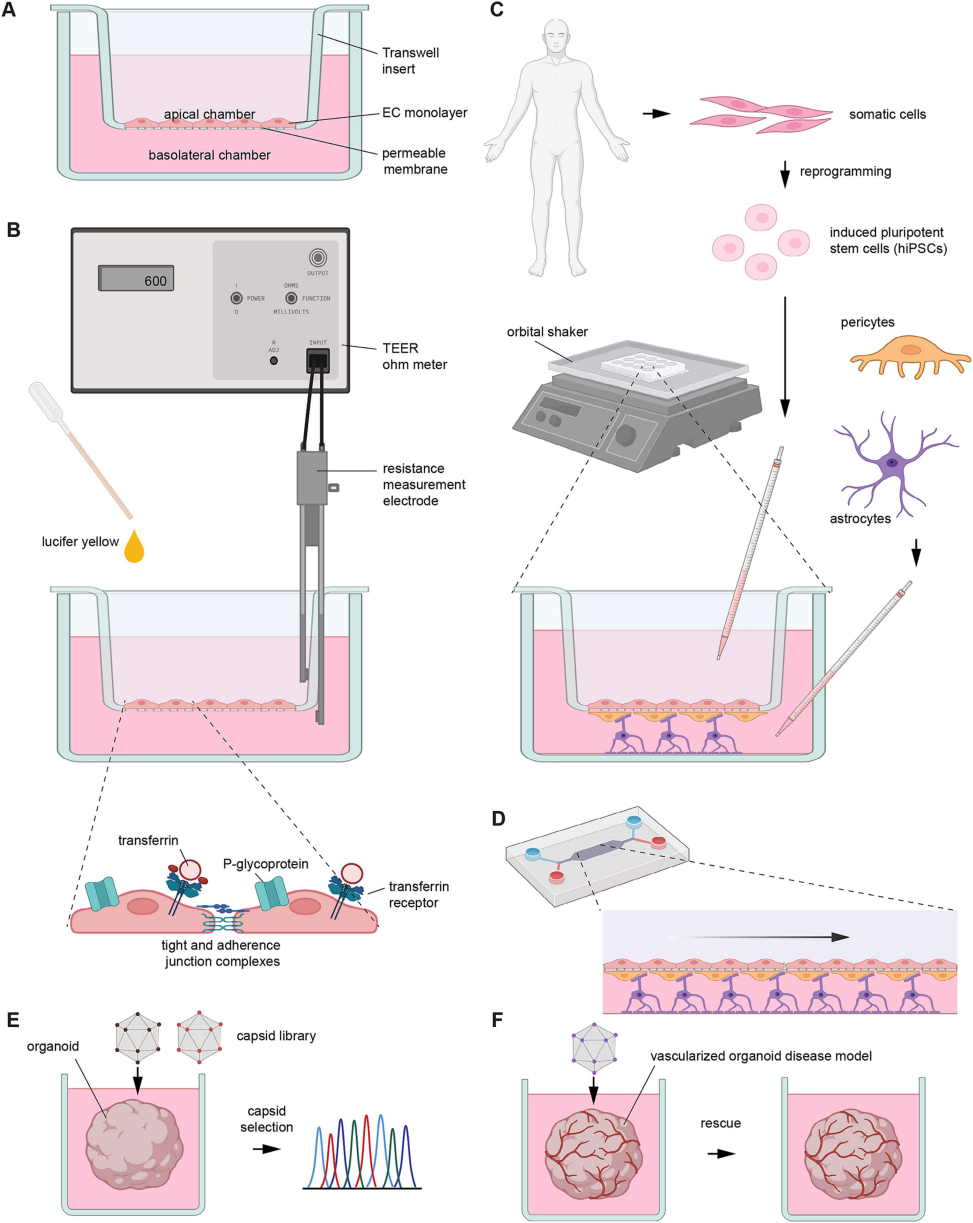
Predicting human BBB penetrance. Illustration depicting key concepts and tools for predicting the human BBB penetrance of rAAV vectors. **(A)** First-generation model of human BBB in a Transwell system. The EC monolayer in this system would typically be based on an immortalized cell line that can acquire EC characteristics, such as hCMEC/D3 cells. **(B)** Orthogonal methods for assessing barrier integrity of the EC monolayer, including measurements of TEER values, passive penetrance of dyes, such as lucifer yellow, and the confocal microscopy-based assessment of the apical-basal polarized distribution of transferrin or P-glycoprotein receptors, or the establishment of cell–cell junctional complexes. **(C)** Advanced human BBB model incorporating, in addition to an EC monolayer, supportive cells such as pericytes and astrocytes. Sophisticated designs source these cells from hiPSCs through terminal differentiation into EC or other cells. Agitation on an orbital shaker is designed to mimic shear forces that exist in blood vessels. **(D)** Advanced implementations of human BBB models aim to recapitulate shear forces more authentically by making use of microfluidic channels coated with EC monolayers. **(E)** Use of human brain organoids for the selection of rAAV vectors with desired tropism. **(F)** Pre-clinical testing of therapeutic rAAV vectors in disease model of vascularized brain organoid. This image was generated with BioRender software (https://biorender.com).

Before a gene therapy can be intravenously administered to humans, it is essential to test how well the vehicle used for its delivery can overcome the human BBB. The classic paradigm for this kind of analysis is the Transwell model, a stacked cell culture system whose upper and lower chambers are separated by a permeable membrane (Figure 3). Its ease of handling made it the go-to platform for early high-throughput screens (Helms et al., 2016). On the other end of the spectrum are highly complex 3D “organ-on-a-chip” cell culture paradigms grown in microfluidic chambers that can recapitulate the physiological shear stress of the cerebral blood flow (Li et al., 2024). While there are strategies to incorporate shear stresses also within Transwell systems using orbital shaking (Kim et al., 2023), microfluidic models may better mimic a vascular microenvironment that can promote authentic gene expression required for forming a tight monolayer. To boost membrane integrity and capture more closely the physiological multicell architecture of the BBB, brain microvascular endothelial-like cells can be grown on feeding layers composed of astrocytes and/or pericytes (Song et al., 2022; Li et al., 2024). Other recent implementations incorporate human induced pluripotent stem cell (hiPSC)-derived cortical neurons (Stone et al., 2019) and microglia for additional authenticity (Chim et al., 2024). Appropriately, this platform is called “BBB-on-a-chip” and recapitulates the physiological characteristics and neuroinflammatory responses of the human BBB (Chim et al., 2024).

At the heart of all BBB model systems is a tight monolayer of endothelial cells (ECs) grown alongside other cells on semipermeable membranes that separate a luminal apical fluid space (mimicking the blood vessel lumen) from a basolateral compartment (mimicking the intracerebral environment) (Helms et al., 2016). The degree of barrier formation can be inferred from transmembrane endothelial electrical resistance (TEER) measurements. Several studies have established an inverse correlation between high TEER and low paracellular permeability (Gaillard and de Boer, 2000). However, this measure of BBB integrity has been shown to be less reliable in low- to mid-range values (below 650 *Ω*/cm^2^) (Thomsen et al., 2021). Additionally, factors including temperature, electrode placement, and measuring device, can influence the TEER measurement, especially for lower TEER values, which can further complicate interpretations (Vigh et al., 2021). Other indirect measures of EC integrity are assessments of the expression and organization of tight junction proteins, the polarized localization of P-glycoprotein efflux transporters, and the expression of the transferrin receptor (Thomsen et al., 2021). More direct assessments of barrier integrity involve measuring the paracellular permeability of small hydrophilic, fluorescent molecules such as sodium fluorescein or lucifer yellow, or high molecular weight tracers such as FITC-dextran (Helms et al., 2016; Li et al., 2024). In addition, rAAV-mediated transduction carried out at 4 °C can limit cell-mediated transcytosis, restricting the passage of materials through an EC layer to paracellular diffusion, thereby providing a measure of leakiness of the cell monolayer (Merkel et al., 2017; Qin et al., 2023).

Human ECs capable of forming tight monolayers can be obtained from several sources. Chief amongst them are primary or immortalized ECs, or ECs differentiated from hiPSCs. Whereas primary ECs most closely resemble the physiological BBB, sourcing them from human specimens can be challenging, and their maintenance in culture can be expensive and complex, limiting their practical use mostly to small-scale experiments. In contrast, immortalized cell lines such as hCMEC/D3 are easily accessible. They are also extensively studied and offer a more reproducible paradigm than the primary cells. However, immortalized cell lines exhibit a higher degree of leakiness for low MW compounds (< 500 Da) and relatively low TEER values (Helms et al., 2016). On the upside, the paracellular permeability of hCMEC/D3 cells for large molecules such as 70 kilodalton dextran is still low, ensuring that transport of rAAV capsid across these artificial barriers requires transcytosis (Song et al., 2022). Several studies have shown how hCMEC/D3 cells can be used in the context of BBB permeability screenings of rAAVs (Eigenmann et al., 2013; Song et al., 2022; Qin et al., 2023; Li et al., 2024). More recently, hiPSCs have emerged as a viable alternative to immortalized cell lines. They exhibit superior membrane integrity manifesting in high TEER values (Lu et al., 2021). However, to date, hiPSC differentiation into authentic ECs remains challenging, evidenced by their tendency to inadvertently acquire transcriptomic profiles similar to epithelial cells (Lu et al., 2021). Parallel to the development of ever more sophisticated BBB assembloids, human organoids are increasingly being deployed for the identification of novel rAAV capsids and the preclinical evaluation of brain-directed gene therapies (Kaiser and Gonzalez-Cordero 2025). In particular, vascularized organoids, whose development is still in its infancy, will be of considerable interest for mimicking the tropism of systemically administered rAAVs.

## Cell type- and region-selective gene therapies and cross-correction

Despite considerable advances in the development of rAAVs for CNS applications, reaching most brain cells in humans following systemic administration of rAAV vectors remains a profound challenge. Even the most promising capsids for this application, such as STAC-BBB, cannot transduce more than 26–65% of neurons in cynomolgus monkeys (Chou et al., 2025b), which have brain volumes that are approximately 20-fold smaller than the human brain (Collins et al., 2010; Croxson et al., 2018). This places a ceiling on the efficacy of rAAV gene therapy designs for the treatment of neurodegenerative diseases that impact the brain broadly, thereby calling for strategies that sidestep this limitation.

One of them is to focus on neurological diseases whose etiologies have a strong regional focus. An example is Huntington’s disease (HD), a dementia characterized by an outsized influence of the striatum on disease progression. HD is a caused by a trinucleotide (CAG) repeat expansion in the huntingtin (*HTT*) gene, causing abnormally long polyglutamine tracts within the HTT protein to build up and poison neurons. How this disease may be treated with an AAV-delivered gene therapy has recently been showcased by what appears to be a groundbreaking advance (reported by UniQure). Specifically, AMT-130, a microRNA targeting the encoded portion of Exon 1 within the *HTT* mRNA, may have slowed disease progression by ~75% in a 3-year time window (Kaiser, 2025). To achieve this feat, the payload was encapsulated within the natural AAV5 capsid, and the gene therapy was administered in a lengthy magnetic resonance imaging (MRI)-guided surgical procedure to six deep brain regions within and near the striatum. The efficacy of this approach may be further enhanced by shifting the payload to capsids which have a more selective tropism for deep brain structures affected in Huntington’s disease. One such capsid, termed AAV-DB-3 was recently reported and shown to transduce 45% of deep brain spiny neurons (Leib et al., 2025).

Another strategy for addressing the delivery limitations of existing rAAV vectors relies on cross-correction (CC). The concept of CC first gained traction for the treatment of lysosomal storage disorders (LSD) (Biffi, 2015). In CC, the subset of cells that can be reached with existing methods is turned into ‘factories’ that secrete a disease-correcting protein, thereby affecting neighboring cells. For instance, functional lysosomal enzymes can be expressed in the liver following rAAV transduction. The liver-secreted enzymes then aid the systemic clearance of accumulated lysosomal substrates (Puzzo et al., 2017; Massaro et al., 2021; Chen N. et al., 2023). A better way to achieve brain-wide CC is to target brain cells. Intravenous delivery of a virus with strong tropism for BECs, AAV-BR1 (Körbelin et al., 2016), carrying the Niemann-Pick C2 gene (*Npc2*), resulted in widespread Npc2 protein in the brain. This alleviated neurological symptoms in a mouse model (*Npc2*−/−) of Niemann-Pick type C2 disease, a genetic lysosomal storage disorder caused by mutations in the *Npc1* or *Npc2* gene (Rasmussen et al., 2025).

A suitable protein target for applying cross-correction to a neurological disease is the APOE-ɛ2 isoform, a naturally secreted APOE variant that can protect against LOAD (Corder et al., 1994). Strong proof-of-concept data in mice validated that this approach can be effective (Rosenberg et al., 2018; Günaydin et al., 2024; Jackson et al., 2024), especially for homozygous APOE-ɛ4 carriers whose risk of developing LOAD is approximately 15-fold increased (Corder et al., 1993; Strittmatter et al., 1993). To achieve a somewhat authentic distribution of the payload-encoded APOE-ɛ2 protein, the expression can be driven by a Willebrand factor A domain containing 3A (VWA3A) promoter that is active in ependymal cells (Jackson et al., 2024). This strategy ensured that APOE-ɛ2 is mostly available in relevant brain areas. When implemented in an AD mouse model (APP/PS1/APOE4 mice), APOE-ɛ2 levels reached 30% of total APOE levels, which led to a significant reduction in Aβ plaque density, synaptic loss and microglial reactivity (Jackson et al., 2024).

A possible limitation of the above cross-correction paradigms may be that mitotic BECs and even ependymal cells—which can serve as reservoirs of latent neural stem cells—can undergo cell divisions, which could dilute the therapeutic capacity of these cells over time. It therefore may be advantageous to target non-dividing neurons and astrocytes for long-term cross-corrective treatments. In cases where disease-correcting proteins are not naturally secreted, protein engineering can be used to enable CC (Puzzo et al., 2017; Massaro et al., 2021; Medici et al., 2022). A recent study investigated the intracerebral administration of a neurotrophic rAAV vector coding for palmitoyl-protein thioesterase 1 (PPT1), a protein deficient in neuronal ceroid lipofuscinosis type 1 (Alam et al., 2025). Specifically, the coding sequence for the Ppt1 protein was N-terminally fused to the endoplasmic reticulum signal sequence borrowed from the human chymotrypsinogen B2 gene, causing secretion of the engineered fusion protein.

Another modification that can enhance CC is the fusion of a protein-of-interest to a sequence that facilitates cellular uptake. CDKL5 is an intracellular protein implicated in a CDKL5 deficiency disorder known as Mucolipidosis Type IV that affects neurodevelopment. In a recent implementation of the CC approach, CDKL5 was fused to an N-terminal immunoglobulin kappa (IgK) signal sequence and a modified cell-penetrating peptide derived from the HIV-1 Tat protein (TATk) to enable both its secretion and subsequent uptake by neighboring cells, respectively (Al-Alawi et al., 2022). The delivery of a PHP. B encapsulated vector coding for Igk-TATk-CDKL5 rescued phenotypical motor and learning deficits, promoted neuronal survival, and improved dendrite morphology in mice lacking CDKL5. It remains to be seen if the IgK-TATk fusion construct can also be adopted for the brain-wide dissemination of other proteins with protective capacity that do not get naturally secreted.

## Studies to watch

Despite early success with AAV9 encapsulated virus vectors, including OA, the intravenous administration of rAAV vectors for the treatment of CNS disorders remains a challenge in the clinic (Reilly et al., 2022). In 2022, two pediatric patients died from acute liver failure after being administered OA, prompting Novartis to update the drug’s label. To date, no clinically approved gene therapy utilizes rAAV vectors whose tropism has been optimized for CNS applications. In fact, very few clinical trials have been undertaken with rAAV vectors that incorporated novel capsids.

A notable exception is a gene therapy aimed at restoring syntaxin-binding protein 1 (STXBP1) expression in patients suffering from STXBP1-related developmental epileptic encephalopathy (DEE). This trial was based on the intravenous injection of CAP-002, a liver detargeted capsid optimized for brain-wide neuronal expression from Capsida Biotherapeutics, and recently reported the death of a child and the termination of the Phase 1/2a SYNRGY study (NCT06983158). Although this setback came as a shock and warning, it seems premature to assume that it will be predictive of the clinical adaptation of other CNS-targeting rAAV capsids. The sequence variances from the fairly well tolerated natural AAV9 capsid are just too small and varied across the most promising CNS penetrant capsids for a prediction to be made based on this one case. Investigations into the root cause of death of the patient, which remains unresolved, are likely to consider also recent data which point toward the capacity of AAV genomes to induce cytotoxicity (Duba-Kiss and Hampson, 2025), including innate immune mechanisms of vector sensing that can lead to p53-dependent cell death, followed by inflammatory responses (Costa-Verdera et al., 2025).

There is considerable momentum in the CNS gene therapy field (Kang et al., 2023; Ling et al., 2023; Xu et al., 2024). In the years to come, if these medicines are to advance to the clinic, this momentum needs to be matched in resources set aside to ease navigating the regulatory landscape. Currently, the path to clinical approvals remains challenging and lacking in precedents that can show the way (Lomash et al., 2025). Hospitals, insurance providers and governments need to work together to generate frameworks that can incentivize the research and sustain getting these medicines to patients. For instance, of the >50 countries whose regulatory agencies have approved OA, approximately a third do not include this treatment in public reimbursement lists, leaving patients to face high cost barriers.

With industry driving much of the innovation and the obvious potential for commercialization, public dissemination of information is often delayed, and it can be difficult to anticipate where critical advances may arise next.

Out of the many interesting lines of investigation that we are aware of, we will highlight two programs that may hold valuable lessons for the clinical translation of rAAV vector-delivered gene therapies.

Lexeo Therapeutics is conducting several AAVrh10-delivered gene therapy trials for APOE-ɛ4 homozygous patients. Their preclinical work on an AD mouse model expressing the human APOE-ɛ4 transgene (APP.PS1/TRE4) found that the expression of the protective APOE-ɛ2 variant leads to a dose-dependent reduction of insoluble Aβ_1-42_ of 78.6% compared to mock-injected mice following intrahippocampal administration (Zhao et al., 2016). Subsequent data collected with African green monkeys established that intracisternal injection of their APOE-ɛ2 gene therapy is an effective administration method for widespread expression in the brain, which led to its advancement to clinical trials (Rosenberg et al., 2018). A press release from October 2024 reported a reduction of CSF tau biomarkers and reductions in global tau PET signals in two-thirds of AD patients (*n* = 15) (Lexeo Therapeutics press release from Oct. 30, 2024). The company is additionally investigating whether the protective gain-of-function Christchurch mutation within the APOE-ɛ2 allele, termed APOE-ɛ2Ch, when administered alongside the APOE-ɛ2 allele, is more effective than the expression of APOE-ɛ2 alone (LX1021) (Günaydin et al., 2024). Finally, a third trial by Lexeo Therapeutics combines expression of APOE-ɛ2 with concurrent suppression of APOE-ɛ4 (LX1020). The results of these trials may have a significant impact on a subset of the population carrying the APOE-ɛ4 risk factor for LOAD, while supporting the use of CC to achieve brain-wide effects.

A common intervention strategy for currently untreatable and invariably fatal prion diseases aims to reduce steady-state levels of the endogenous cellular prion protein (PrP^C^) which serves as the substrate for the conversion to a disease-associated conformer (Minikel et al., 2020). One approach to lowering PrP^C^ expression pursued by Sangamo Therapeutics is to block the transcription of the prion gene (*PRNP*) with customized zinc finger repressors (ZFRs) (Chou et al., 2025a). In a proof-of-concept study in mice, which made use of STAC-BBB as the delivery vehicle, a reduction in steady-state PrP^C^ levels of 56% was reported. Whereas untreated mice succumb to disease in about 150 days after inoculation with disease-causing prions, the treatment extended survival in prion-infected mice to 500 days post-inoculation (dpi). Importantly, the intervention was effective even when administered to mice at 120 dpi, a late timepoint when they already begin to display prion disease symptoms. Next, Sangamo assessed the PrP^C^-lowering capacity of their therapy in cynomolgus macaques. Although the switch from rodents to NHPs resulted in a lesser reduction in PrP^C^ levels across various brain regions, *Prnp* mRNA levels were profoundly lowered (by 65–98%) in neurons, the cell type most affected in prion diseases. In addition, the clinical lead, ZFR ST-506, suppressed *PRNP* expression in human iPSC-derived neurons by >90% (Chou et al., 2025b). The first clinical trial investigating the intravenous infusion of the promising STAC-BBB capsid delivering ZFRs is expected to commence in 2026 (Chou et al., 2025a; Chou et al., 2025b). In addition, the company has partnered with Genentech to develop an analogous therapy that targets the tau gene (*MAPT*), which has shown potent dose-dependent reductions of *MAPT* expression across CNS regions in both mice and NHPs (Zeitler et al., 2024).

## Conclusion

Addressing the challenge of safe and efficient CNS delivery of gene therapies represents an urgent unmet need. This need is increasingly being met by fast-paced research focused on rAAVs as the delivery vehicle of choice. So often when a research domain becomes deeply investigated, a myriad of intricacies come to light that stand in the way of easy solutions. It is now increasingly being appreciated how even subtle changes to rAAV capsids can have profound impacts on tropism and cross-species potency. There also is heightened awareness of the need to consider age, delivery route, and the many shades of seropositivity when conducting clinical studies. The recent death of a child who participated in a trial of a novel rAAV vector has been a tragic wake-up call. Despite these setbacks, it is apparent that the epitaph will not be written for this delivery platform any time soon. Too much is at stake, and there are many hopeful signs that this technology is coming of age. Among them are promising capsids that can be purified to high titers and purities, dock to known cellular uptake receptors, are peripherally detargeted and potent across species. As in other competitive fields, some opportunities for synergy are not yet realized because methods are slow to become standardized. This plays out in several ways, including a reality of groups deploying divergent methods for the assembly, purification, titration, characterization, and administration of rAAV vectors, thereby precluding a meaningful comparison of the relative potencies of existing rAAV vectors. What has become apparent is that no capsid will shine in all domains. Consequently, rather than focusing optimization on a specific trait, capsids need to be viewed as decathlon athletes that are not the best at any individual challenge but excel when faced with all of them. Machine learning is beginning to help in this domain. A major advantage over small molecule-based therapies is the modular nature of the task. Like the field of computing, challenges of this kind are ideally suited to creating synergy through collaboration and incremental advances in several domains. In fact, just as we were preparing this manuscript for submission, a report described a Muscovy duck AAV isolate, AAV.div3A, that evades the mammalian immune system, thereby offering hope to individuals who are AAV seropositive or require repeat dosing for improved efficacy of a future rAAV-delivered gene therapy (Loeb et al., 2025). The significance of progress toward repeat rAAV administrations cannot be overstated. Solutions in this area would ease the need to succeed in the development of rAAV vectors that can achieve the intended therapeutic goal upon one-time administration. Advances in this domain would not only open the door to consecutive dosing with a given treatment (Spathis et al., 2025) but would also ensure that patients can benefit from advances to gene therapies for a given condition or can be treated for multiple conditions with distinct gene therapies in their lifetime. In addition to building this capacity on stealth AAVs that naturally evade immune responses, this objective can be achieved through insights gained from studying the molecular underpinnings that drive immunological responses to rAAV vector exposure. Perhaps, the most hopeful aspect of this concerted effort toward better rAAV capsids and regimens that allow repeat vector administration is the knowledge that once available tools and methods meet the high bar, these solutions may be useful for delivering potent gene therapies for the treatment of many CNS disorders.
